# Analysis of nestin protein in the aqueous humor as biomarker of open angle glaucoma

**DOI:** 10.1016/j.heliyon.2022.e09753

**Published:** 2022-06-19

**Authors:** A. Pulliero, A. Izzotti, L. Pastorino, S. Gandolfi

**Affiliations:** a Department of Health Sciences, University of Genoa, Via A. Pastore 1 Genoa, Italy; b UOC Mutagenesis and Cancer Prevention, IRCCS Ospedale Policlinico San Martino, Largo Rosanna Benzi, 10, Genoa, Italy; c Department of Experimental Medicine, University of Genoa, Italy; d Department of Informatics, Bioengineering, Robotics and Systems Engineering, University of Genova, Via all’ Opera Pia 13, 16145 Genoa, Italy; e Ophthalmology Unit, Department of Biological Biotechnological and Translational Sciences, University of Parma, Parma, Italy

**Keywords:** Primary open angle glaucoma, Early diagnosis, Open quartz crystal microbalance (QCM), Chemical coating on gold surface, Quartz sensor

## Abstract

Primary open angle glaucoma (POAG) is a progressive optic nerve degeneration, leading to irreversible visual damage. Alterations of the aqueous humor (AH), the biological fluid filling both the anterior and the posterior chambers of the eye, play a pathogenic role in POAG. AH protein composition is altered during glaucoma progression. Nestin protein was found to be differentially expressed in the AH of glaucomatous patients compared to unaffected matched controls. Methods: Nestin was analyzed by an open quartz crystal microbalance (QCM) in the AH of 21 glaucomatous patients compared to nine unaffected controls. The surface of the electrode used in the QCM was coated with an analyte-specific recognition layer. Results: Positive nestin values were recorded in the AH collected from POAG patients; negative values of nestin detection were obtained by analyzing the AH collected from non-POAG glaucomatous patients and unaffected controls. Conclusion: The present study proposes and validates a new clinically applicable approach to analyze biological markers in AH for POAG diagnosis.

## 1 Introduction

Glaucoma is the leading cause of irreversible blindness. The Primary Open Angle (POAG) is the most prevalent subtype among Caucasians and afro-caribbeans, and it likely affects more than 90 million people worldwide. An increased intraocular pressure (IOP) is still the main risk factor for diagnosis and progression, albeit 6% of the general population shows an elevated IOP without any clinically detectable disease (Ocular hypertension OH) [1].

POAG is often diagnosed once an extensive amount of visual damage had occurred [2]. Early diagnosis is then pivotal in preventing irreversible visual disability.

An impaired proteomics in the aqueous humor has been described in samples withdrawn from eyes affected by POAG “in vivo” [3]. Neural proteins, mostly released from the damaged trabecular meshwork, have been detected in glaucoma, while being undetectable in samples collected from age-matched healthy controls [4]. Nestin is a neural filament protein expressed in dividing cells of the central nervous system during the developmental stages and in activated and proliferating glial cells [5]. Nestin was found to be differentially expressed in the AH and in the TM of glaucomatous patients compared to unaffected matched controls [6]. The levels of nestin in AH correlated with IOP and VF defect in the individual patients [4]. Identification of proteome alterations in glaucomatous AH is useful to explore the pathogenesis of this disease and to identify new molecular targets for glaucoma therapy. Nestin, PRKA and APR2 are dramatically increased in AH of glaucomatous patients as compared to age matched controls [6, 7]. The presence of nestin in other structures of the ocular anterior segment, i.e., the cornea and crystalline, was previously reported [8, 9]. Identification of proteome alterations in glaucomatous AH is useful to explore the pathogenesis of this disease and to identify new molecular targets for glaucoma therapy.

Should a real time detection, obtained by using a simple and “clinician-friendly” technique, be feasible, then nestin could represent an interesting molecule to investigate as a potential biomarker of the disease.

The hereby presented study was in fact designed to develop a diagnostic biochip to identify and quantify nestin (together with specific glaucoma proteins) in the aqueous humor obtained from human eyes. The obtained results help in the measurement of nestin in the AH of POG patients in real time using a simple and practical approach easily transferable to the clinical practice.

## 2 Materials and methods

### 2.1 Patients recruitment

The study had a cross-sectional case-control design. Both patients and controls, all Caucasian, underwent ocular surgery for therapeutic purposes. AH samples were obtained from POAG patients (cases) and age-related cataract patients (controls) prior to trabeculectomy and cataract surgery, respectively.

The study was approved by the Ethical Committee of the University of Genoa (n.12102010).

### 2.2 Inclusions and exclusion criteria

Case samples were collected from 18 POAG patients (8 males, 10 females), 3 non-POAG glaucoma patients, and 8 controls, for a total of 29 subjects. The main elements for POAG diagnosis were papilla morphology, IOP values, and visual field analysis. Exclusion criteria were the presence of any other ocular, systemic, or neurological diseases other than POAG-related optic-nerve damage. The age of POAG patients was 74.9 ± 3.10 years (means ± SE). All patients underwent a Humphrey 30-2 computerized visual field examination (750 Humphrey Field Analyzer II; Humphrey Ind., San Leandro, California) 14–32 days before surgery. To evaluate visual field damage, the Glaucoma Staging System (GSS 2) was used [10]. All patients underwent daily tonometry curves (every 2 h between 8:00 a.m. and 8:00 p.m.) the week before surgery. This procedure was performed by the same physician using Goldmann tonometry.

The mean IOP values (mean ± SD) were 28.6 ± 3.6 mmHg for glaucoma patient and 17.2 ± 1.8 for cataract controls.

The mean stage of glaucomatous visual field defects in PAOG patients was 3.4 ± 1.5 (mean ± SD) according to GSS Glaucoma Staging System classification (min1, max 5).

Glaucoma types and age of patients are reported in Table 1.

**Table 1:** Subjects characteristics.

Code Subject	Pathology	Age	Calculated Nestin value ng/ml AH
UA 52	POAG	89	1013.9
UA 272	POAG	90	545.9
UA 281	POAG	85	3829.0
UA 159	POAG	86	7915.4
UA 146	POAG juvenile	54	0
UA 190	POAG	61	6647.9
UA 167	POAG	60	1669.7
UA 147	POAG	81	7221.9
UA 172	POAG	87	2955.9
UA 76	POAG	90	3476.0
UA 275	POAG	63	1112.6
UA 235	POAG	76	6086.5
UA 70	POAG pseudoexfoliatio	89	0
UA 61	POAG	90	1133.6
UA 229	POAG	82	1920.0
UA 36	POAG juvenile	61	0
UA 208	POAG	82	2410.7
UA 141	POAG	81	7007.9
UA 121	POAG	60	6944.1
UA 151	POAG	80	197.6
UA 120	POAG	98	401.4
UA 19	Cataract	80	0
UA 10	Cataract	86	0
UA 49	Cataract	69	0
UA 51	Cataract	83	0
UA 227	Cataract	76	0
UA 23	Cataract	87	0
UA 40	Cataract	74	0
UA 33	Cataract	52	0

All enrolled subjects were treated in accordance with the Declaration of Helsinki. The study was approved by the ethical Committee of the University of Genoa and San Martino Hospital on December 7^th^ 2010, issue number 170/2010.

### 2.3 Aqueous humor sampling

For both cases and controls, a 100 mL intravenous injection of mannitol was administered before surgery, and eyes were treated with peribulbar anesthesia (bupivacaine hydrochloride 5%). Pupils were dilated with phenylephrine 10% and tropicamide 1%. Eyelids and surrounding skin were swabbed with disinfectant, and AH (50–80 μL) was aspirated by corneal puncture, which involved inserting a 26-gauge needle into the AC just before surgery. AH sample aliquots were immediately stored in a deep freezer (−80 °C) until quartz crystal microbalance (QCM) measurement was performed.

### 2.4 Quartz sensors

**Figure 1:**
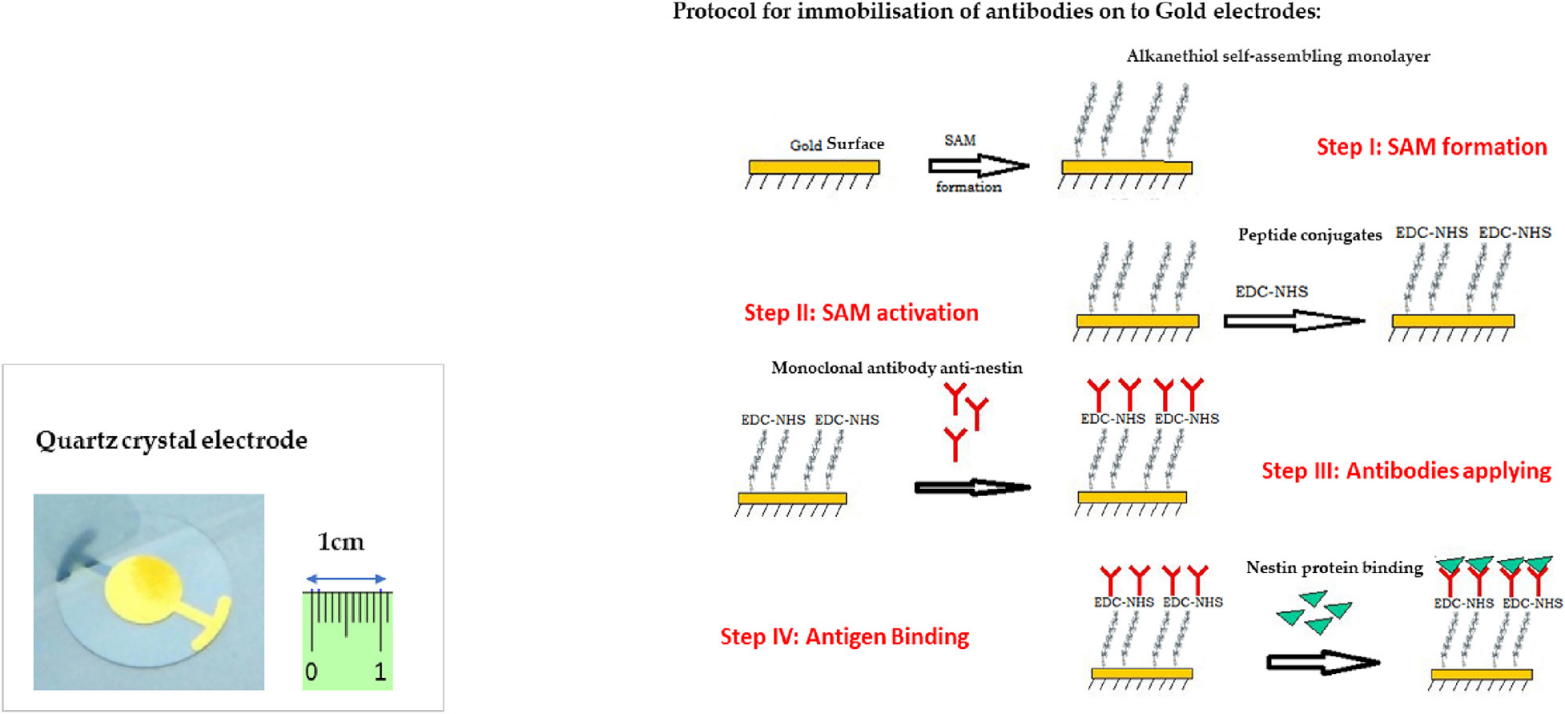
**(a)** Quartz crystal electrode used for the quartz crystal microbalance (QCM), with black diameter of 13.9 mm; frequency of 10 MHz; gold electrodes (thickness 200 nm); and nominal sensitivity down to 4.42 × 10 ^−9^ gHz^−1^cm^−2^**. (b)** Description of the immobilization of antibodies onto gold electrodes. The first step was the preparation of the alkanethiol self-assembling monolayer (SAM). After functionalization by SAM, the electrode was washed with ethanol to remove unbound thiols. The second step consisted of activating the monolayer coated gold surface by exposure to EDC/NHS mixture, and then applying monoclonal antibody anti-nestin 1 mg/mL (dilution 1:100) to the functionalized gold surface. The last step was applying the nestin antigens for protein binding testing.

Quartz crystals are generally used for oscillation frequencies between 0.5 and 300 MHz. Cut quartz crystal sensors were used with a fundamental oscillation frequency of 10.0 MHz, diameter of 13.9 mm, gold electrodes (thickness 200 nm) deposited by physical vapor deposition method, and nominal sensitivity of 4.42 × 10 ^−9^ gHz^−1^cm^−2^. The quartz crystal resonance frequency is highly sensitive to mass changes on the crystal surface, meaning that a mass deposited onto the surface of the crystal causes a decrease in its resonance frequency. The resulting decrease in resonance frequency is proportional to the mass of the deposited analyte. Oscillation quartz functionalized with specific biomolecules has been widely used in mass sensors for the detection of clinically relevant analytes [10, 11, 12]. The quartz electrode used for the QCM in our study is shown in Figure 1 (a).

### 2.5 Immobilisation of antibodies on to gold electrodes.13

Quartz crystals were first cleaned with H_2_SO_4_ 956% at 150 °C for 30 min, followed by washing with ultrapure water. The crystals were the dried under N_2_ flux and further pre-processed under a UV 254 nm hood for 15 min. The preparation of self-assembled alcanethiol (SAM) is constituted by an 11-mercapto-1-undecanoic acid (MUA) and 11-mercaptoundecanoal (MU) acid. The quantity of 4 mM of MUA and 1 mM of MU are stately prepared separately in ethanol and kept at ambient temperature.

Subsequently, equal volumes of solution were immediately added to the gold surface, which was exposed to the solution for 12 h (overnight). The SH group of the thiol reacts rapidly with the Au of the piezoelectric crystal and binds to the substrate via the sulfur atom, and the COOH group of the thiol binds to the primary amino group –NH2 of the peptide. After the functionalization with SAM alkanethiol, the electrode was washed with ethanol to remove unbound thiols.

The next step was preparation of 1-Ethyl-3-[3-dimethylaminopropyl] carbodiimide hydrochloride (EDC) and N-hydroxysuccinimide (NHS). Peptide conjugates consisted of 100 mM of NHS in Millipore water and 400 mM of EDC in Millipore water, separately. The aliquots were stored separately at −20 °C. To activate the SAM, the monolayer coated gold surface was exposed to the EDC/NHS mixture for 10 min by mixing an equal volume of 100 mM NHS and 400 mM EDC (v/v). The excess of EDC/NHS solution on the electrode was rinsed once with phosphate buffered saline (PBS) buffer. Monoclonal antibody anti-nestin 1 mg/ml (dilution 1:100) (Abcam, ab18102) was applied to the functionalized gold surface for 30 min. After 30 min, the antibody solution was rinsed from the surface with PBS buffer. A quantity of 1 M ethanolamine hydrochloride, pH 8.5 (stored in the refrigerator at 4 °C), was exposed for 7 min to the gold electrode to terminate the unreacted sites. At this stage, the antibody was successfully coupled and ready for the assay. To avoid the possibility of nonspecific binding, Tween20 detergent was added to the standard phosphate buffer saline (PBS) solution while preparing the nestin antigens. To set up the standard reference curve, commercial nestin protein was diluted to a concentration of 1.5 μg/mL and 0.15 μg/mL in PBS with Tween 20 to eliminate non-specific bonds and left in contact with the transducer surface for 15 min (Figure 1 a, b).

The samples were then washed in PBS to remove molecules that were not specifically bonded. The gold surface should be clean and further pre-processed under a UV 254 nm hood for 15 min. The AH sample equal to 20 μL was placed and left in contact with the surface of the transducer for 10–15 min.

### 2.6 Quartz crystal microbalance, QCM

The QCM consists of a gravimetric method based on quartz crystals that exploits the piezoelectric properties of the quartz crystal. The QCM device consists of the quartz electrode, the oscillator, and the frequency counter.

When an electric field is created on the QCM, an oscillation is created in the resonance mode. The decrease of the resonant frequency depends on the mass change in the system. When the surface of the quartz crystal encounters the sample, the resonant frequency decreases due to the adsorption of the analyte.

The change in resonance frequency was measured by the Sauerbrey equation [12]:

$$-\Delta \text{F}\left(\text{Hz}\right)=\frac{2{\text{F}}_{\text{0}}^{2}}{\text{A}\sqrt{\text{μqρq}}}\Delta \text{m}\left(\text{ng}\right)$$

and related to the mass of the bound antigen (Δm, ng) and to the thickness of the layer (Δt, nm), where F_0_ is the resonance frequency of the quartz crystal oscillator, A represents the area of the gold electrode, μq is its shear modulus and ρq is the quartz density.

We used a QCM to measure the antigen–antibody interaction occurring on the surface of the quartz crystal, which is within an oscillating circuit in which mass variations cause a decrease in the resonance frequency of the crystal [13]. Given that the quartz oscillation frequency is inversely proportional to the mass deposited on the functionalized electrode, this system can provide qualitative and quantitative measurements relating to the presence or absence of the analyte [14]. For the QCM calibration, the following procedure was applied. Calibration was conducted with an impedance probe by running a procedure including phase correction, open, short, and load measurements. Using the calibration line obtained, a measurement range was established to identify the exact concentration of the proteins of interest by referring to the data already obtained on their concentrations in AH samples from pathological and control subjects [6]. The different amounts of proteins present in the available samples were then compared to establish a measurement cut-off. Two QCM readings were made, one before placing the sample, and one after incubation with the AH sample and washing step.

### 2.7 QCM analysis program

The use of the analysis program applied to the QCM was adapted to the data reading program using Arduino electronic support and modified for the specific application of the QCM, in which the innovation is the use for the early diagnosis of glaucoma. This program can easily be used by the operator and, via Excel masks, allows the patient's registration, the clinical characteristics, and the measured nestin protein values to be recorded, and identification of a threshold value for the presence or absence of the protein. A graphical user interface that controls the impedance analyzer was implemented in MATLAB. The graphical user interface monitors the resonance frequency over time and the corresponding resistivity. The graphical user interface allows the measurement to be established, and the data recording to be started and stopped. A conversion model was set up to calculate the quantitative mass adsorption based on the frequency shift.

The dissipation values were calculated from the resistance measurement. Before starting the measurement, a wide range frequency sweep was performed to extrapolate the required equivalent circuit parameters using the integrated circuit analysis software in the impedance analyzer. For resonances relative to the value of the high-quality factor (Q), the effect of C_0_ represents a slight offset of the absolute resonant frequency. Therefore, by eliminating the effect of C0 and of the parasitic circuit elements deriving from the liquid cell, the compensation of the device was obtained. Additionally, an algorithm was used to find the relevant resonant frequencies. the interpolation capabilities and corresponding tracking search markers of the impedance analyzer, led to the implementation. The sweep frequency of the software was detected every 1s corresponding to a refresh rate of 1 Hz (Figure Supp 1).

These complex preparation steps were performed to set up the device to be used by clinicians. The surgeon is only requested to withdraw the AH sample and to drop it on the QCM sensor.

## 3 Results

**Figure 2:**
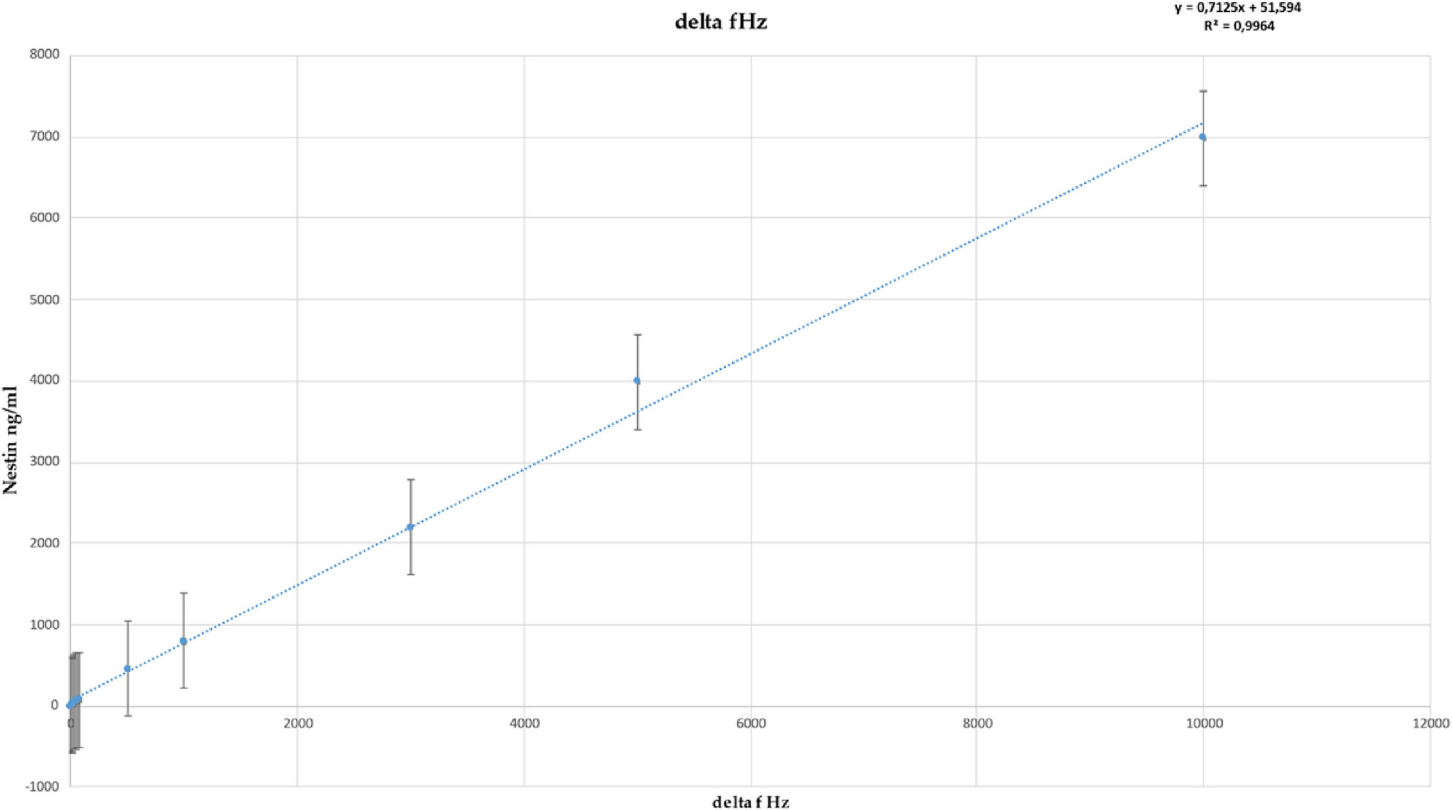
Dose response curve of established nestin concentrations used as a standard reference curve.

In POAG patients, the nestin value calculated was positive and the quantity was always higher than 100 ng. In non-POAG patients (controls) the nestin value was negative and no nestin in AH was detected (Table 1). The QCM program shows that the time-dependent resonance frequency was modified according to the binding of nestin on the biosensor and the consequent changes occurring in its weight compared to the empty biosensor. Based on the changes occurring in the frequency of biosensor oscillation, the calculation of the bound nestin in ng was undertaken according to the Sauerbrey equation. The value of the protein mass detected is displayed after the calculation. The threshold was arbitrarily fixed at 100 ng, although the method is sensitive enough to detect nestin amounts that are 10 times less (10 ng), as inferred from the calibration curve (Figure 2).

The calibration curve was established by analyzing five independent experiments. The standard deviation of the five results obtained for each dilution is reported in Figure 2. Accordingly, a total of 40 independent analyses were performed to establish the reference curve. The interval of this curve was selected in line with nestin concentrations found in AH samples of POAG patients. Higher concentrations could hamper QCM oscillation and detecting concentration near or below 1 ng could decrease the specificity of the analysis for POAG detection.

The 100-ng high threshold for clinical application was arbitrarily used to increase the specificity of the test. The display window turns to red when this threshold is reached, otherwise turning to green. Accordingly, the only action required by the clinical is to look at the displayed color.

**Figure 3:**
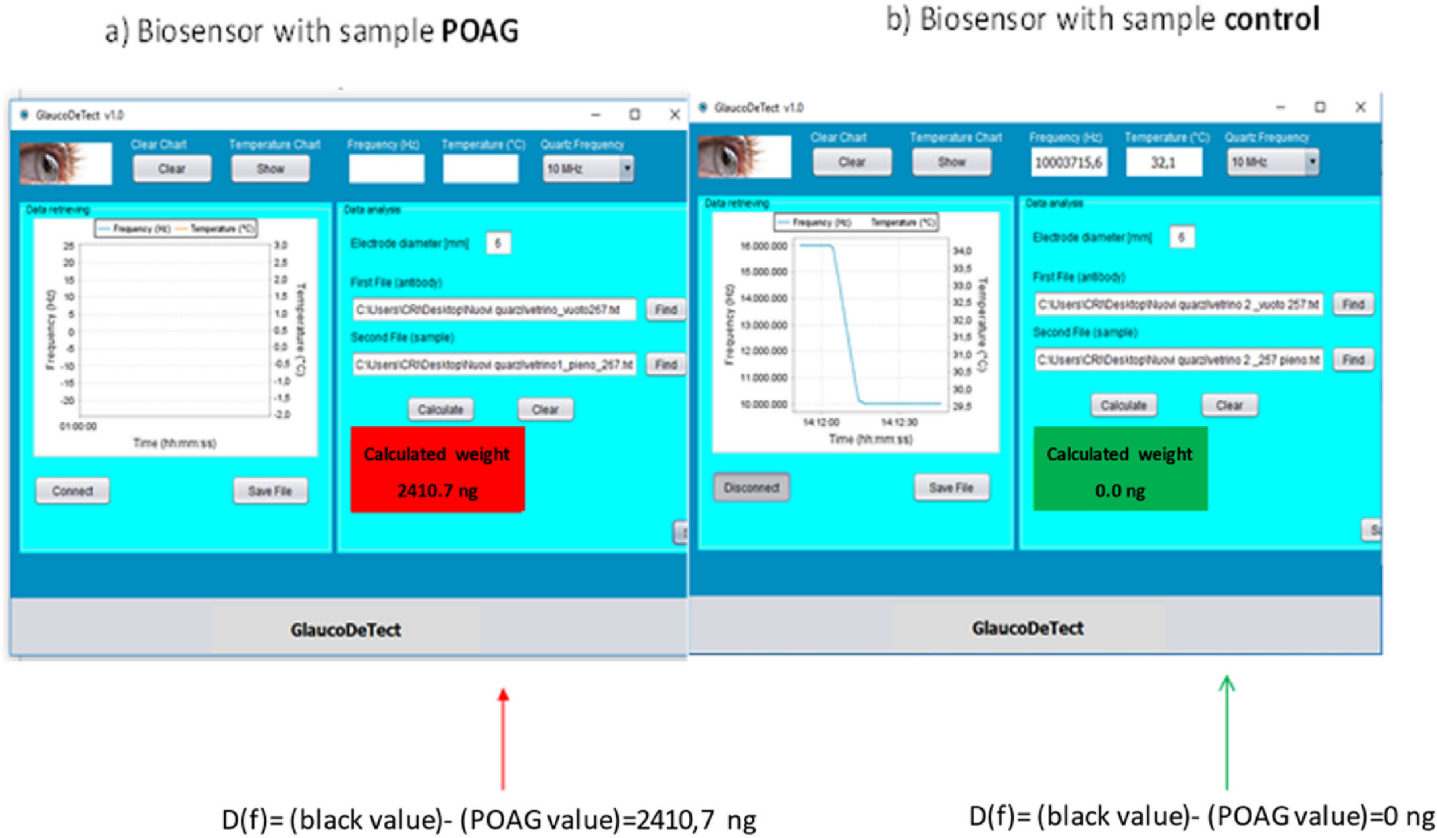
Figures 3a and 3b shows examples of the analyses of two different subjects. The display window turns to red for a positive sample (left), and green for a negative sample (right), depending on the presence or absence of nestin protein.

Figures 3a and 3b shows examples of the analyses of two different samples, one negative and one positive.

**Figure 4:**
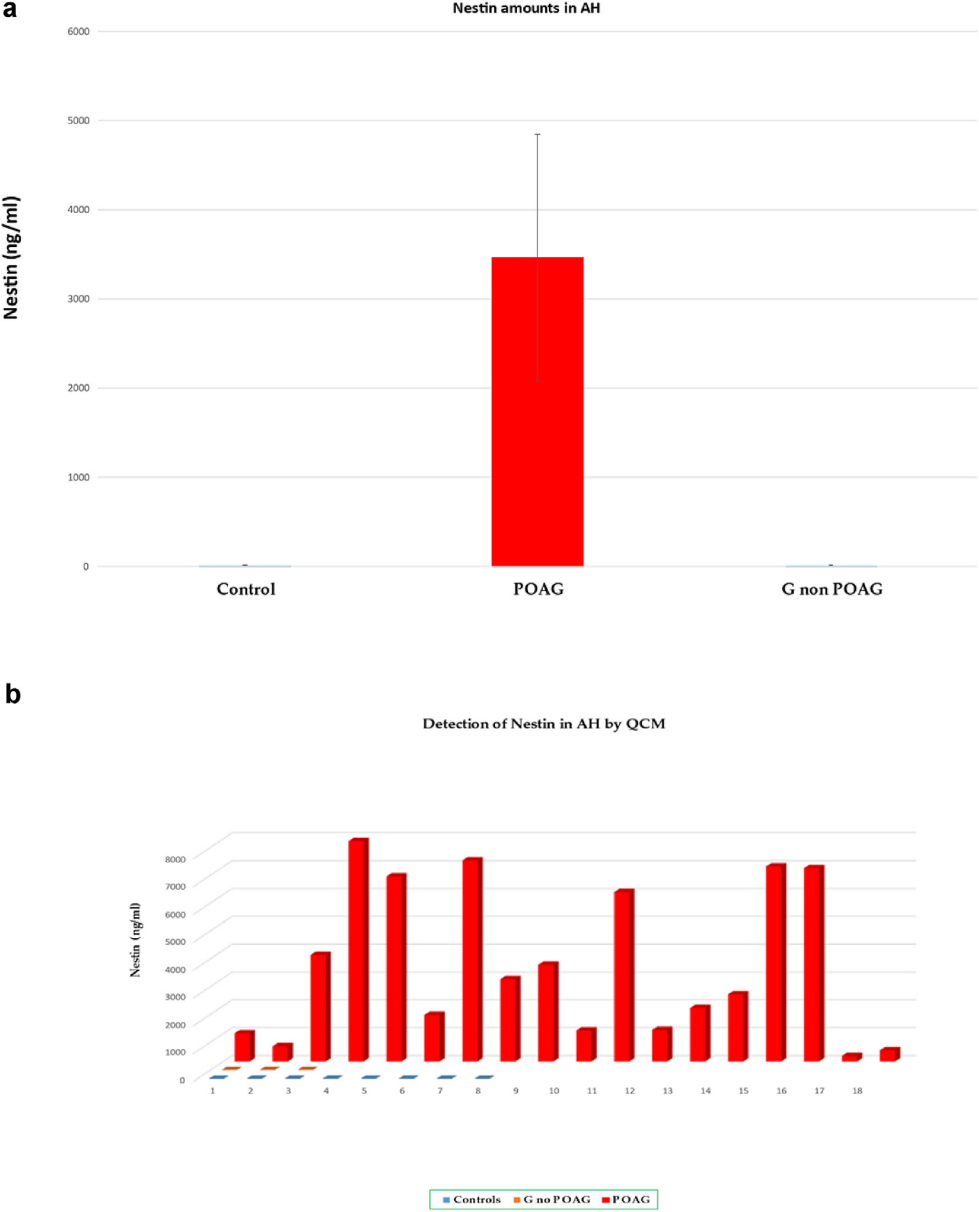
a. Quantification (mean ± SD) of nestin protein by QCM in the AH of (a) Control (glaucoma free subjects); (b) POAG (primary open angle glaucoma (POAG) patients; (c) glaucomatous (G) patients affected by other glaucoma types than POAG (columns from left to right). b. **Single Nestin values detected in** (a) Control (glaucoma free subjects) (n = 7, blue columns); (b) POAG (primary open angle glaucoma (POAG) patients (n = 18, red columns); (c) glaucomatous (G) patients affected by other glaucoma types than POAG (n = 3, green columns). Positive nestin values were recorded in all POAG patients; in controls and non-POAG glaucomatous patients, nestin was not detected by QCM in AH.

Recorded nestin values in the AH of POAG patients are well above the threshold of 100 ng, spanning up to very high values, i.e., more than 5000 ng when the disease is very advanced (Figure 4a).

We calculated the presence of nestin protein in nanograms. Positive nestin values were recorded in all POAG patients, whereas in controls and non-POAG glaucomatous patients, negative values of nestin were observed in AH (Figure 4b). This difference is statistically significant (*p* < 0.001), as evaluated by nonparametric (Mann–Whitney U test) tests (Figure 4b).

## 4 Discussion

In the hereby presented study, we were able to quantify a neural protein, nestin, in the AH of POAG patients, by using a novel detection system, easier and faster than other currently available methods.

Proteome alterations has been widely detected in AH proteome by several studies [15]. However, nestin is particularly interesting because of its peculiar pathogenic role in POAG pathogenesis.

Nestin expression in the anterior ocular chamber of POAG patients has been already detected by immunohistochemistry, Western blot and antibody microarray both by our previous studies [7] and by those of other authors [16]. For the first time in our knowledge, the herein presented paper provides evidence that nestin can be detected in AH by QCM.

Nestin is a glia activator involved in neurodegenerative diseases and glaucoma. Nestin expression specifically occurs in neural tissues with reference to those undergoing glial activation and glia proliferation. Under physiological conditions, nestin is expressed at a low level in the TM. However, nestin expression dramatically increases in the damaged TM [4, 15]. TM has a mixed embryological origin, with both endodermal and neuroectodermal origins. This peculiar situation is unique for the TM, which is a complex organ composed of components arising from different embryo layers (ectodermal and mesodermal) [17]. The connection between oxidative damage, the main pathogenic factor of POAG, and glial activation as induced by nestin, has been previously well documented [18]. Furthermore, in experimental glaucoma of the rat, elevated IOP induces Müller glial cell activation as manifested by nestin expression [16]. Therefore, it is not surprising that TM, once affected by any external insult (glaucoma included), will express nestin in detectable amounts both in the tissue and the AH. In fact, the concentration of nestin, in the AH of glaucomatous subjects, is 7.2-fold with respect to controls [4].

The minimal amount of TM proteins released into the AH requires the use of sensitive methods for their detection, as previously discussed in a review paper [4]. The method presented in this paper represents an approach for intercepting TM proteins in the AH that is simple, reliable, and readily transferable to the clinic, thus avoiding the high complexity and costs of using antibody microarrays or Gas chromatography–mass spectrometry methods (GCMS) methods. Piezoelectric sensors use a QCM to measure antibody–antigen interaction by monitoring a decrease in resonant frequency due to the mass increase when an antibody, immobilized on a quartz crystal Au sensor surface, captures an antigen from a sample such as AH. Piezoelectric sensor assays are simple, rapid, sensitive, and typically only require a single antibody to carry out the analyses. The medical applicability of the peptide for immobilization of an antibody on a gold electrode for the detection of proteins in human body fluids has been already demonstrated [14, 19]. The present study demonstrates for the first time the feasibility of detecting nestin in human AH with high sensitivity and specificity using QCM piezoelectric sensors. In this diagnostic device, the antigen–antibody interaction occurs on the surface of the quartz crystal, which is within an oscillating circuit in which mass variations cause a decrease in the resonant frequency of the crystal [13]. A QCM responds to changes in mass on the electrode surface and has a sensitivity of the order of nanograms. Using a calibration line, we established a measurement range to identify the exact concentration of the proteins of interest by referring to previously obtained data presented in the literature regarding their concentrations in AH samples from pathological and control subjects [4, 5, 6]. The versatility of the QCM in combination with a multitude of surface chemically modified sensors provides a flexible system. As an example, a thymine-imprinted thin-film-coated QCM sensor was developed to recognize DNA fragments [20]. Quartz crystal microbalance dissipation was used to measure the real-time kinetics and total uptake of protein adsorption onto supported lipid bilayers [21]. QCM-based sensors have been used in the detection of matrix viscoelastic changes due to the treatment of breast cancer cells with cytoskeleton-targeting drugs such as CytoD and Y27632 [22].

In the present study, more traditional methods, as GCMS and ELISA, proved less sensitive than QCM in detecting nestin in the AH, the volume of the sample being insufficient or the total amount of proteins being too minimal (data not shown). In fact, the methods presented in this study were developed to solve these problems. Further, comparative analysis was performed using ELISA and QCM with 500 ug of nestin reference standard proteins. Such a large amount is clearly easily detectable by ELISA, but QCM oscillations were blocked, making the analysis unfeasible. These results indicate that nestin analysis in AH requires a specific approach as represented by the proposed device. Indeed, the specificity of nestin as glaucoma biomarker depends on its finding in the aqueous humor. The detection of nestin in blood would be unsensitive and unspecific; indeed nestin is modified in neurological diseases involving neural cells activation and proliferation in central nervous, system such as glioblastoma.

AH specimen collection is an invasive and a difficult method for patients. Patients reported in our paper underwent ocular surgery for therapeutic purposes (glaucoma therapy, i.e. trabeculostomy and trabeculectomy), or cataract patients (lens insertion, controls). AH sampling procedure has been practiced anyway independently from the purpose of our study. Accordingly, one chance is to use AH for QCM nestin detection instead of wasting this fluid after ocular surgery. AH sampling is going to be easier in the next future using new minimally invasive device [23]. At this regard, QCM would be an interesting method to be used because the minimal volume (30–40 ul) of samples required and the advantage of avoiding any purification of extraction steps. Indeed, after collection AH can be deposited directly on the QCM quartz crystal in such a minimal amount that is the necessary quantity to cover the sensor surface thus allowing reading to take place.

In conclusion, our study confirms the presence of significant amount of nestin in the AH of patients affected by chronic glaucoma. Nestin is expressed in damaged trabecular meshwork during POAG course. Undamaged ocular and neural tissue do not express Nestin. The finding that nestin can be detected only in AH collected from POAG patients and not from controls or non POAG patients (i.e. those not having a damaged TM) is in line with POAG pathogenesis. The QCM technique seems adequately sensitive to detect the protein even at low concentrations and into small-volume samples. Being the technique fast, user-friendly, and feasible in a clinical setting, it is worthwhile exploring its potential in testing and validating nestin as a clinical biomarker both in the diagnosis and in the progression of glaucoma.

## Institutional review board statement

The study was conducted according to the guidelines of the Declaration of Helsinki and approved by the ethical Committee of the San Martino Hospital on December 7^th^ 2010, issue number 170/2010.

## Informed consent statement

Informed consent was obtained from all subjects involved in the study.

## Declarations

### Author contribution statement

Alessandra Pulliero: Performed the experiments; Wrote the paper.

Alberto Izzotti: Conceived and designed the experiments; Wrote the paper.

Laura Pastorino: Contributed reagents, materials, analysis tools or data.

Stefano Gandolfi: Analyzed and interpreted the data.

### Funding statement

This research did not receive any specific grant from funding agencies in the public, commercial, or not-for-profit sectors.

### Data availability statement

Data will be made available on request.

### Declaration of interest statement

The authors declare no conflict of interest.

### Additional information

No additional information is available for this paper.

## Appendix A Supplementary data

Suppl 1 Suppl 1

## References

[1] Kapetanakis V.V., Chan M.P., Foster P.J., Cook D.G., Owen C.G., Rudnicka A.R. (2016). Global variations and time trends in the prevalence of primary open angle glaucoma (POAG): a systematic review and meta-analysis. Br. J. Ophthalmol..

[2] Giuffrè G., Giammanco R., Dardanoni G., Ponte F. (1995). Prevalence of glaucoma in a population. Acta Ophthalmol. Scand..

[3] Chowdhury U.R., Madden B.J., Charlesworth M.C., Fautsch M.P. (2010). Proteome analysis of human aqueous humor. Invest. Ophthalmol. Vis. Sci..

[4] Adav S.S., Jin Wei J., Yap Terence Y., Ang B.C.H., Yip L.W., Sze S.K. (2018). Proteomic analysis of aqueous humor from primary open angle glaucoma patients on drug treatment revealed altered complement activation cascade. J. Proteome Res..

[5] Tezel G. (2013). A proteomics view of the molecular mechanisms and biomarkers of glaucomatous neurodegeneration. Prog. Retin. Eye Res..

[6] Izzotti A., Longobardi M., Cartiglia C., Saccà S.C. (2010). Proteome alterations in primary open angle glaucoma aqueous humor. J. Proteome Res..

[7] Izzotti A., Ceccaroli C., Longobardi M.G., T: Micale R., Pulliero A., La Maestra S., Saccà S.C. (2015). Molecular damage in glaucoma: from anterior to posterior eye segment. The MicroRNA role. MicroRNA.

[8] Wilson S.E., Sampaio L.P., Shiju T.M., Carlos de Oliveira R. (2020). Fibroblastic and bone marrow-derived cellularity in the corneal stroma. Exp. Eye Res..

[9] Park T., Curran T. (2020). Requirement for Crk and CrkL during postnatal lens development. Biochem. Biophys. Res. Commun..

[10] Shons A., Dorman F., Najarian J. (1972). The piezoelectric Quartz immunosensor. J. Biomed. *Mater. Res*..

[11] Bastiaans G.B., Edmonds T.E. (1988).

[12] Vaughan R.D., O'Sullivan C.K., Guilbault G.G. (1999). Sulfur based self assembled monolayers (SAM's) on piezoelectric crystals for immunosensor development Fresenius J. Anal. Chem..

[13] Yang Y., Liu Q., Gao Z., Qiu X., Meng L. (2015). Data fault detection in medical sensor networks. Sensors.

[14] Tombelli S., Mascini M., Turner A.P.F. (2002). Improved procedures for immobilisation of oligonucleotides on gold-coated piezoelectric quartz crystals. Biosens. Bioelectron..

[15] Izzotti A., Centofanti M., Saccà S.C. (2012). Molecular diagnostics of ocular diseases: the application of antibody microarray. Expert Rev. Mol. Diagn.

[16] Xue L.P., Lu J., Cao Q., Hu S., Ding P., Ling E.A. (2006). Müller glial cells express nestin coupled with glial fibrillary acidic protein in experimentally induced glaucoma in the rat retina. Neuroscience.

[17] Tripathi B.J., Tripathi R.C. (1989). Neural crest origin of human trabecular meshwork and its implications for the pathogenesis of glaucoma. Am. J. Ophthalmol..

[18] Ogundele O.M., Omoaghe A.O., Ajonijebu D.C., Ojo A.A., Fabiyi T.D., Olajide O.J., Falode D., Adeniyi P.A. (2014). Glia activation and its role in oxidative stress. Metab. Brain Dis..

[19] Shang Y., Singh R., Mernaugh A.R., Chisti M.M., Zeng X. (2011). Immobilization of a human epidermal growth factor receptor 2 mimotope-derived synthetic peptide on Au and its potential application for detection of herceptin in human serum by quartz crystal microbalance. Anal. Chem..

[20] Diltemiz Emir S., Hür D., Ersöz A., Denizli A., Say R. (2009). Designing of MIP based QCM sensor having thymine recognition sites based on biomimicking DNA approach. Biosens. Bioelectron..

[21] Yorulmaz S., Jackman J.A., Hunziker W., Cho N.J. (2016). Influence of membrane surface charge on adsorption of complement proteins onto supported lipid bilayers. Colloids Surf. B Biointerfaces.

[22] Bianco M., Vergara D., De Domenico S., Maffia M., Gaballo A., Arima V. (2018). Quartz crystal microbalance as cell-based biosensor to detect and study cytoskeletal alterations and dynamics. Biotechnol. J..

[23] Toris C.B., Gagrani M., Ghate D. (2021). Current methods and new approaches to assess aqueous humor dynamics. Expet Rev. Ophthalmol..

