# Supplementary figures and images for: Analysis of nestin protein in the aqueous humor as biomarker of open angle glaucoma
Source: Heliyon. 2022 Jun 19;8(6):e09753. doi: 10.1016/j.heliyon.2022.e09753 (PMC9249827; doi:10.1016/j.heliyon.2022.e09753)

## Slide 1
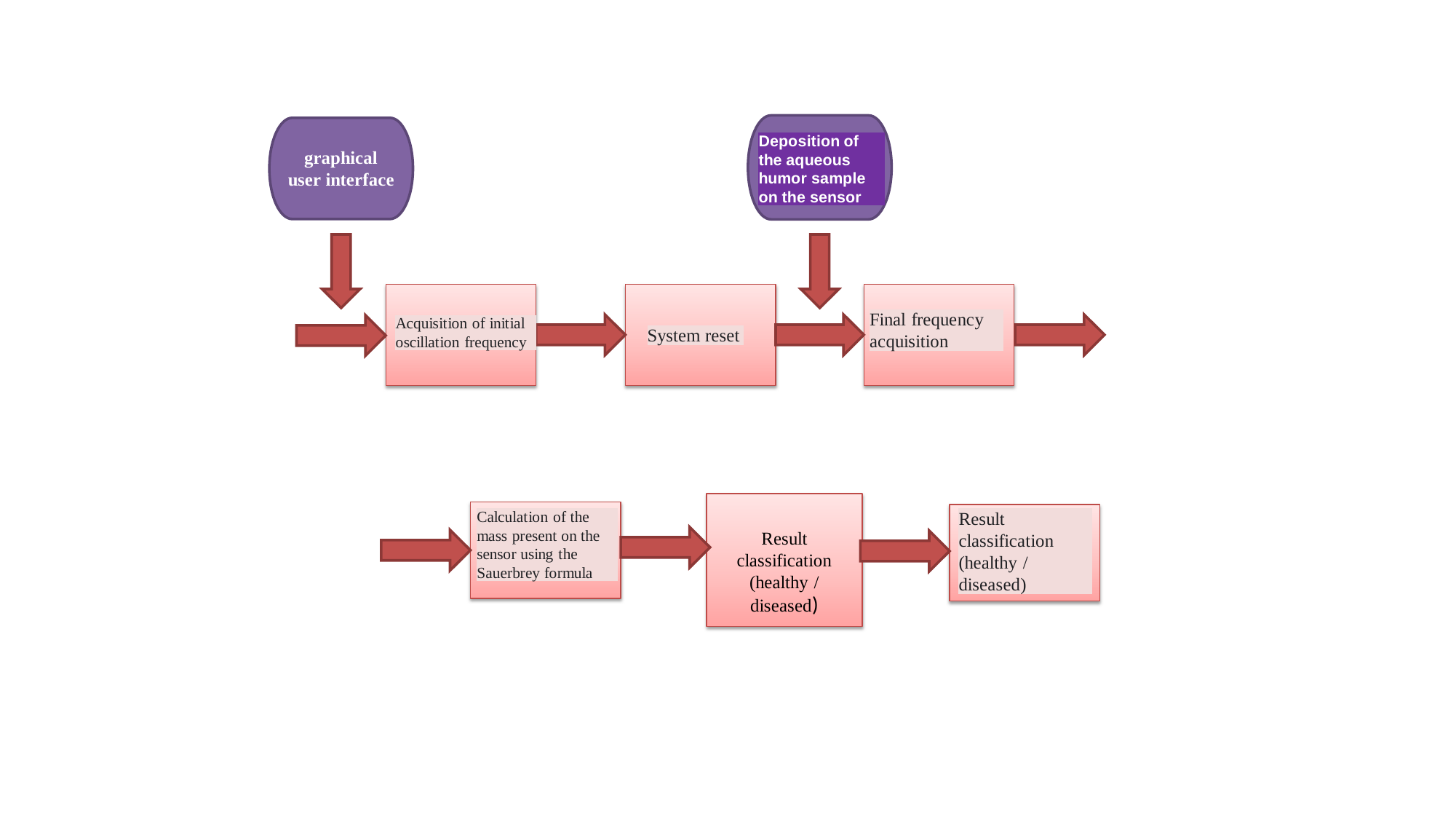

Supplement: Suppl 1 [file mmc1.pptx]
